# Rapid Access Diagnostics for Asthma (RADicA): protocol for a prospective cohort study to determine the optimum series of investigations to diagnose asthma using conventional and novel tests

**DOI:** 10.1136/bmjopen-2024-083908

**Published:** 2024-10-26

**Authors:** Clare S Murray, Stephen Fowler, Sarah Drake, Ran Wang, Hannah J Durrington, Hannah Wardman, Laura Healy, Miriam Bennett, Andrew Simpson, Emma Barrett, Stephen A Roberts, Angela Simpson

**Affiliations:** 1 Faculty of Biology, Medicine and Health, School of Biological Sciences, Division of Immunology, Immunity to Infection & Respiratory Medicine, The University of Manchester, Manchester, UK; 2 Manchester Academic Health science Centre, Manchester University NHS Foundation Trust, Manchester, UK; 3 School of Sport, Exercise and Rehabilitation Sciences, University of Hull, Hull, UK; 4 Centre for Biostatistics, Institute of Population Health, The University of Manchester, Manchester, UK

**Keywords:** Asthma, Respiratory Function Test, Pulmonary Disease, Chronic Obstructive, Paediatric thoracic medicine

## Abstract

**Abstract:**

**Introduction:**

The diagnosis of asthma is often based on characteristic patterns of symptoms in the absence of an alternative explanation, resulting in over and under diagnosis. Therefore, diagnostic guidelines usually recommend including confirmation of variable airflow obstruction. Some recommend using a sequence of objective tests; however the tests used, the specific cut-off values and the specified order are yet to be validated. We aimed to determine the optimal cut-off values and series of investigations to diagnose asthma. We also explore the potential for novel tests of small airways function and biomarkers, which could be incorporated into future diagnostic pathways.

**Methods and analysis:**

The Rapid Access Diagnostics for Asthma study is an observational study of 300 symptomatic patients with ‘clinician-suspected asthma’ and healthy controls (aged ≥3 to <70 years), recruited from primary and secondary care in Greater Manchester, UK. Symptomatic participants will undergo four core visits and one optional visit. Participants will complete two baseline visits and undergo a series of established (spirometry, bronchodilator reversibility, exhaled nitric oxide, home peak flow monitoring and bronchial challenge testing) and novel tests. Following visit 2, participants will receive monitored medium-dose inhaled corticosteroid therapy for 6–8 weeks, after which they will return for repeat testing. Patients will be diagnosed with asthma by ‘expert panel’ opinion (minimum two respiratory specialists) on review of all data (excluding novel tests) pre and post treatment. Healthy controls will attend two visits to establish reference intervals and calculate repeatability coefficients for novel tests where there is a lack of evidence on what threshold constitutes a ‘normal’ set of values. The primary end point is to determine the optimum diagnostic pathway for diagnosing asthma.

**Ethics and dissemination:**

The study was approved by Greater Manchester East Research Ethics Committee (18/NW/0777). All participants or parents/guardians are required to provide written informed consent and children to provide written assent. The results will be published in peer-review journals and disseminated widely at conferences and with the help of Asthma and Lung UK (www.asthmaandlung.org.uk).

**Trial registration number:**

ISRCTN11676160.

**STRENGTHS AND LIMITATIONS OF THIS STUDY**

- Participants have symptoms suggestive of asthma and are not using inhaled corticosteroids (ICS).
- There is no reference standard for the diagnosis of asthma, we have used a panel of experts (including at least two senior asthma specialists) to evaluate all evidence, including history, physical examination and results from conventional tests (ie, excluding novel tests) pre and post treatment with ICS, to determine participant outcome.
- Any proposed new pathway or test will require future validation.
- Any findings will not be applicable to heavy smokers or those with other significant comorbidities.

## Introduction

 There is currently no single ‘gold standard’ test to confirm (or refute) a diagnosis of asthma. Asthma is a clinical diagnosis based on a characteristic pattern of symptoms, signs and test results.^1^ In a Canadian study of a large cohort of adults with a recent diagnosis of asthma, careful re-evaluation ruled out the diagnosis of asthma in one-third of the cases.^2^ This likely reflects a combination of asthma remission and over diagnosis, but in a minority, an alternative serious cardiorespiratory condition was diagnosed. The European Asthma Research and Innovation Partnership identified improving the diagnosis of asthma among their 15 key research priorities.^3^ Due to concerns about the over and under diagnosis of asthma, UK experts have developed comprehensive guidance on the diagnosis of asthma incorporating objective tests, on behalf of the National Institute of Health and Care Excellence (NICE).^2 4 5^ The algorithm incorporates the sequential use of five measures of lung function and inflammation, each applied as a dichotomous variable: (1) spirometry, (forced expiratory volume in one second (FEV_1_)/forced vital capacity (FVC)), (2) bronchodilator reversibility (BDR), (3) fractional exhaled nitric oxide (FeNO), (4) peak expiratory flow variability (PEFv) and (5) bronchial hyper-responsiveness testing (BHR) to methacholine or histamine. A minimum of two tests must be positive to confirm the asthma diagnosis. In children, only the first four tests are included in the algorithm. Other diagnostic pathways have also been developed both in adults^6 7^ and in children^8^ and all show considerable heterogeneity.

It is clear that there is a scarcity of data in the diagnostic efficiency of all of the current asthma tests.^15^,^8^ In addition, most published data are from adults, with very few data available from children. Both the accuracy and the ideal sequence of these tests recommended in any of the guidelines remains unknown, and the impact on patient care has not been tested prospectively. The British Thoracic Society/Scottish Intercollegiate Guideline Network states in their current asthma guidelines that there is ‘an urgent need for diagnostic accuracy studies and implementation research to confirm, prospectively, the diagnostic accuracy of retrospectively derived algorithms and to define the optimal approach to making a diagnosis’.^1^ Thus, there is a need to test published diagnostic algorithms to understand their impact on patient care and to identify whether they can be improved by comparing alternative sequences of tests and/or alternative thresholds for positive tests to optimise asthma diagnosis now—a new ‘gold standard’.

Many asthma diagnostic guidelines advocate the use of a trial of treatment, usually in the form of low-dose to medium-dose inhaled corticosteroids (ICS), for those who can either not complete the tests, do not have tests available to them, or for whom tests are inconclusive.^1 5^ Although having steroid-responsive airways disease (SRAD) does not necessarily equate to an asthma diagnosis,^9^ having a biomarker for such a phenotype is useful in its own right.

Current guidelines^5^,^8^ focus on large airway physiology and fail to consider the significant contribution of the small airways in airflow limitation in asthma. Tests of small airways function (eg, forced oscillation technique (FOT), multiple breath washout (MBW))^10^,^13^ and biomarkers of airways inflammation in exhaled breath (particles in exhaled air (PExA), volatile organic compounds (VOCs)) may have a useful role in asthma diagnosis and identification of SRAD.^14^,^17^ These tests all require further validation to establish their potential role in asthma diagnosis.

### Aims and objectives

The primary aims of this study are to determine the optimum diagnostic pathway for asthma and for SRAD, based on conventional (large airway) and novel (small airways and biomarkers) tests, to inform evidence-based recommendations for asthma diagnosis. Optimum cut-off values to rule in asthma will be established for each test. Secondary aims include:

1. Evaluate the performance of NICE asthma diagnostic algorithm and compare performance with new proposed algorithm. Test other international algorithms for comparison (European Respiratory Society (ERS), Global Initiative for Asthma (GINA)).
2. Identify the best predictor(s) of response to ICS from measurements taken at baseline or after early treatment with ICS (after 1–3 weeks of treatment—core visit 3 (CV3)).
3. In healthy volunteers, establish reference intervals and calculate repeatability coefficients for novel tests where there is a lack of evidence on what threshold constitutes a ‘normal’ set of values.
4. Identify the profile of biomarkers in VOCs and PExA which best predict asthma diagnosis.

## Methods and analysis

### Patient and public involvement

The study design, visit content and length were adapted following consultation with patient focus groups facilitated by the Public Programmes Team at the National Institute for Health and Care Research (NIHR) Biomedical Research Centre (BRC) Manchester (VOCAL; www.wearevocal.org). Patients and public have ongoing input into the development of patient information sheets, posters, other patient facing materials and protocol amendments. VOCAL and Asthma and Lung UK (www.asthmaandlung.org.uk) will assist with dissemination of results to the participants and wider public. Focus groups of local general practitioners also gave input into the study design and in particular the recruitment strategy.

### Study design

This is a large prospective cohort study of people with suspected asthma (ie, symptoms of cough, wheeze, chest tightness and breathlessness, not currently receiving ICS treatment), who will undergo a full evaluation with all standard diagnostic tests of airway function, along with some novel tests, both before and after a course of usual asthma therapy (ICS), followed by evaluation of asthma diagnosis by an ‘expert panel’. Participants will undergo four core visits and one optional visit. Recruitment commenced in May 2019. We aim to recruit up to 600 adults and children (300 symptomatic (150 adults:150 children) and 300 healthy controls).

**Figure 1:**
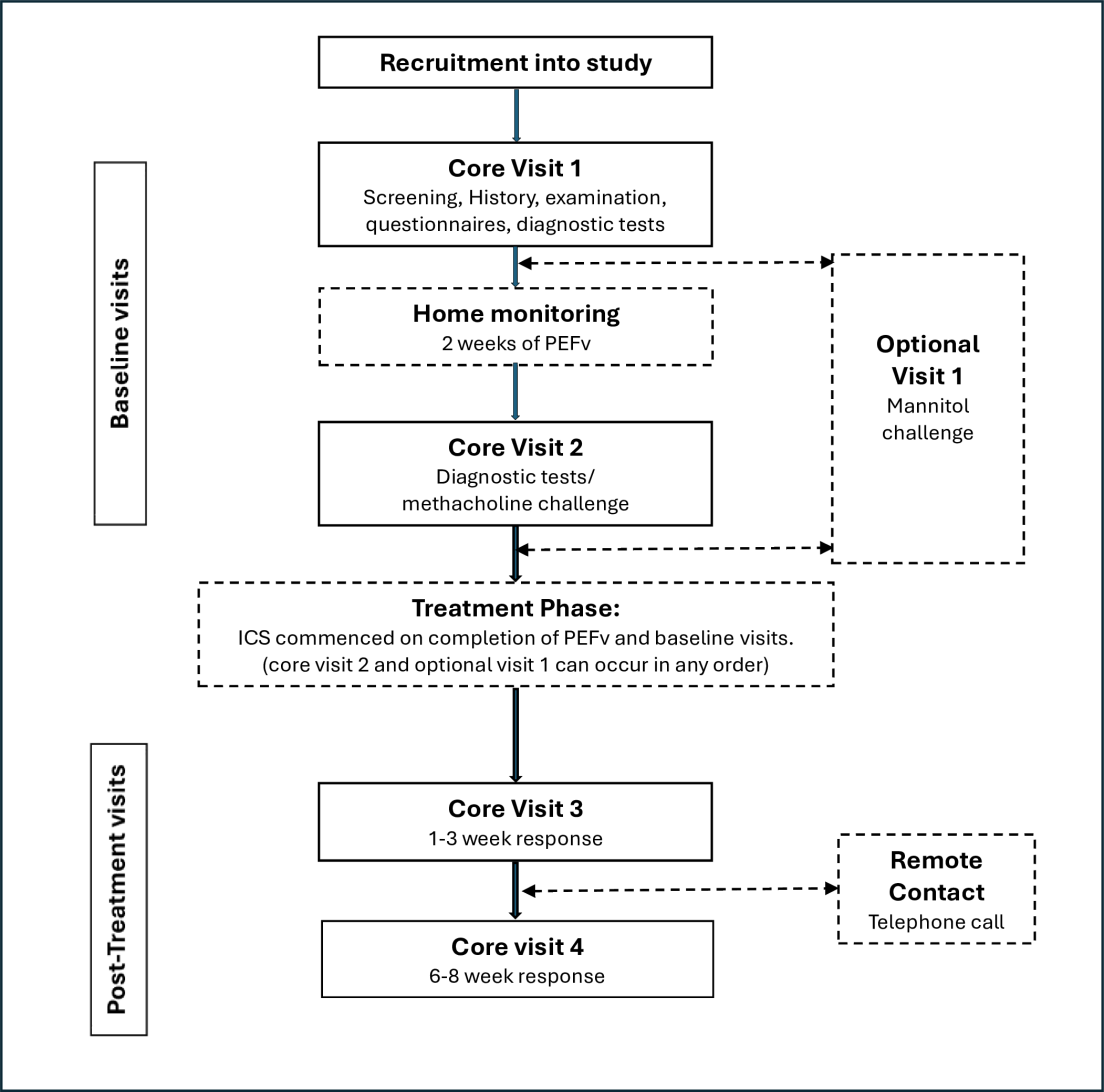
Study design schematic for symptomatic participants. ICS, inhaled corticosteroids; PEFv, peak expiratory flow variability.

The first two visits (CV1 and CV2) plus the optional visit 1 are termed ‘baseline visits’ and will be completed before the patient is commenced on a trial of ICS. The last two visits (CV3 and CV4) are ‘treatment monitoring visits’ and will be performed following initiation of ICS treatment. A study design schematic for the patient group is presented in figure 1.

In addition, age-matched and gender-matched asymptomatic healthy controls will be recruited to assess normal reference ranges and repeatability of novel tests. Participants from the healthy control group will attend two visits: visit 1 (baseline visit) and visit 2 (reproducibility visit).

Table 1 shows the tests to be conducted at each visit for both symptomatic and healthy control groups.

**Table 1:** Study schedule of tests completed at each visit

	Symptomatic group					Healthy group	
	Baseline			Steroid response		Baseline	Follow-up
	CV1	CV2	Optional visit 1	CV3	CV4	Visit 1	Visit 2
Time	Day 0	CV1+14–42 days	Anytime between CV1 and start of treatment	CV2+7–21 days	CV2+42–98 days	Day 0	+7–84 days
Approximate visit duration	2.5 hours	1.5 hours	1 hour	1 hour	2 hours	2 hours	1 hour
Clinical examination	^☑^				^☑^	^☑^	
ACQ-5	^☑^	^☑^	^☑^	^☑^	^☑^		
FeNO	^☑^	^☑^		^☑^	^☑^	^☑^	^☑^
AO	^☑^	^☑^	^☑^	^☑^	^☑^	^☑^	^☑^
VOC	^☑^	^☑^		^☑^	^☑^	^☑^	^☑^
MBW	^☑^	^☑^		^☑^	^☑^	^☑^	^☑^
PExA	^☑^	^☑^		^☑^	^☑^	^☑^	^☑^
Spirometry	^☑^	^☑^	^☑^	^☑^	^☑^	^☑^	^☑^
BDR	^☑^					^☑^	
BCT_(Meth)_		^☑^			^☑^		
BCT_(Mann)_			^☑^				
AO (post BCT/BDR)	^☑^	^☑^	^☑^		^☑^	^☑^	
SPT	^☑^ *					^☑^	
Eos	^☑^ *			^☑^ †	^☑^	^☑^	
PEFv	^☑^ ‡						

☑ indicates tTest completed at this visit.

* Can be completed at Core Visit V2 as alternative if needed.

† In adults and offered in children>12 years.

‡ Given to subjects at end of visit 1 to be collected at visit 2.

ACQ-5, Asthma Control Questionnaire—5; AO, airways oscillometry; BCT_(Mann)_, mannitol bronchial challenge test; BCT_(meth)_, methacholine bronchial challenge test; BDR, bronchodilator reversibility; CVcore visitEos, blood eosinophil levels; FeNO, fractional exhaled nitric oxide; MBW, multiple breath washout; PEFv, peak expiratory flow variabilityPExA, particles in exhaled air; SPT, skin prick test; VOC, volatile organic compounds

### Study setting

Symptomatic subjects will be recruited from primary and secondary care in Greater Manchester, UK. Patients presenting to their physicians with symptoms suggestive of asthma, not currently taking preventer medication, with no previous formal diagnosis, can be referred for eligibility assessment. In addition, healthy volunteers (control group), from the same geographical area, will also be recruited using local advertisement and self-referral into the study. After the recruitment of half the healthy volunteers is completed, the second half will be recruited preferentially to balance age and gender with the symptomatic cohort.

### Target population

#### Symptomatic group

Inclusion criteria (all of the following):

1. Males or females ≥3 years and <70 years.
2. Clinical suspicion of asthma from referring healthcare professional (eg, general practitioner).
3. One or more symptoms in keeping with asthma (ie, cough, wheeze, chest tightness and/or breathlessness).
4. Capable of giving informed consent or where under 16 years attends with parent or legal guardian who can give consent.

Exclusion criteria (any of the following):

1. Recent ICS (used within previous 4 weeks) and/or oral steroid treatment (within the previous 4 weeks).
2. Current/former tobacco smokers with a history of more than 10 pack-years of smoking.
3. Other relevant comorbidities (eg, known lung disease).
4. Antibiotic treatment within previous 2 weeks (recruitment can be deferred).
5. Pregnancy.

#### Healthy control group

Inclusion criteria (all of the following):

1. Males or females ≥3 years and <70 years.
2. Capable of giving informed consent or where under 16 years attends with parent or legal guardian who is able to give consent.

Exclusion criteria (any of the following):

1. Diagnosis or repeat prescription of asthma treatment past or present.
2. Significant respiratory, cardiac or other medical comorbidity.
3. More than one course of antibiotics for chest infection in the last 12 months.
4. Pregnancy.
5. Current/former smokers with a history of more than 10 pack-years of smoking.
6. Recent antibiotic treatment for any cause within previous 4 weeks.
7. Active symptoms of rhinitis (with 2 weeks).

### Study procedures

#### Symptomatic group

##### Core visit 1

Following consent, participants will receive a standardised consultation (including full clinical history, symptom questionnaires and examination). The clinician will then record the initial clinical impression (documented as ‘high probability,’ ‘intermediate probability’, ‘low probability’ and ‘alternate diagnosis more likely’). Participants coded ‘alternate diagnosis more likely’ will be discussed with the supervising specialist physician and withdrawn from the study and referred back to their general practitioner (GP) or referring physician or directly into a clinical service for further investigation.

All other participants will continue in the study and complete a series of tests (see table 1).

Participants will be trained to use a personal electronic peak expiratory flow metre and instructed to complete two times per day measurements for 2 weeks. A salbutamol inhaler will be provided for symptom relief.

If significant concerns about delaying ICS treatment are raised during the baseline visit, a decision can be made to start ICS immediately, with the participant subsequently completing CV3 and CV4 only.

##### Core visit 2

Participants will complete a series of tests (see table 1). The PEFv monitor will be collected, and measurements downloaded. ICS will be commenced.

Initiation of ICS: Education on inhaler technique using an In-Check DIAL G16 Inhaler Training Device (Clement Clarke, UK) and use will be given. Patients will be commenced on 6–8 weeks of ICS treatment with fluticasone propionate (≥16 years old, 250 µg two times per day; <16 years, 100 µg two times per day). If the technique is satisfactory, an Accuhaler (GlaxoSmithKline, UK) will be issued with a digital inhaler compliance device to monitor adherence (e.g. INCA; Vitalograph, Ireland). An option of Evohaler (GlaxoSmithKline, UK) with spacer device (Volumatic; GlaxoSmithKline, UK) will be available for all young children and in the event of any participant not being able to use the dry powder inhaler satisfactorily.

##### Core visit 3

All participants will attend an early follow-up visit after 1–3 weeks of ICS treatment for clinical assessment and objective tests (table 1).

##### Remote contact

Brief telephone contact will be made to all participants to check compliance and symptoms between CV3 and CV4.

##### Core visit 4

All participants will attend a final visit after 6–8 weeks (with allowance up to maximum of 14 weeks in case of difficulties attending, eg, holidays) of ICS treatment for clinical assessment and objective tests (table 1).

When participants have completed visit 4, participants will be informed as to the opinion of the ‘expert panel’ and whether they should continue with ICS treatment. This, along with a copy of all the standard test results, will be forwarded to their GP and referring physician (if different from the GP).

##### Optional visit 1

Prior to the start of ICS, an optional visit will be available to complete tests as detailed in table 1, unless the participant was unable to perform reliable spirometry in a previous visit or it was deemed in the participant’s best interest not to delay further the initiation of ICS. This visit will not be completed within 72 hours of the other baseline visits.

### Asymptomatic healthy group

#### Visit 1

Following consent, participants will complete a series of tests, as detailed in table 1.

#### Visit 2

1–12 weeks later, participants will complete a series of tests, as detailed in table 1.

### Concomitant medications

Where possible, all participants will be asked to withhold the following medications prior to each visit: (1) short-acting beta-2 agonists for 8 hours, (2) ICS for 12 hours (symptomatic group at visits 3 and 4), (3) smoking for 1 hour, (4) caffeine for 8 hours and (5) antihistamine 72 hours (prior to skin pick testing and mannitol challenge only).

### Adverse events

Any adverse events will be recorded. Any serious adverse events will be notified to the study sponsor and the ethics board within 24 hours of observation of the event.

### Measurements

Clinical history and demographics will be recorded at the initial visit: height, weight, ethnic origin, age and smoking history. Participants will complete a set of objective tests (details below) at each visit following study standard operating procedures (available on request; table 1 shows the schedule of events).

*FeNO* is measured according to the manufacturer’s instructions (NIOX VERO, Circassia, UK). Briefly, participants perform an exhalation to residual volume (RV), followed by an inhalation through the device filter to total lung capacity (TLC). Participants are then instructed to make a controlled exhalation for 10 s at a standardised flow rate (50 mL/s±10%), guided by a visual animation.

*Spirometry* is measured with the JAEGER Vyntus PNEUMO (Vyaire Medical, USA), in accordance with the American Thoracic Society/ERS guidelines.^18^ Briefly, the participant is instructed to breathe tidally, then inhale to TLC followed by a maximal exhalation to RV. A minimum of three technically acceptable measurements (ie, free of artefact, slow starts and coughing) are required. The best two measurements are required to be within 5% of each other for the test to be valid. In children, a visual animation using the Vyntus PNEUMO software is used to encourage optimal technique.

*BDR* is measured by repeating spirometry 15 min after 400 µg inhaled salbutamol via large volume spacer. Airway oscillometry will also be repeated post spirometry.

*Methacholine challenge* will be carried out using the Vyntus APS Nebulizer system with integrated dosimeter (Vyaire medical, USA) and a quadrupling dose protocol.^19^ Baseline spirometry is measured and repeated 90 s after each inhalation of methacholine. The challenge is stopped when either a 20% fall in FEV_1_ is observed or the maximum cumulative methacholine dose has been administered. Airway oscillometry will also be carried out before and at the end of the challenge.

*Mannitol challenge* is carried out using a dry power delivery device (Osmohale, Pharmaxis, Australia) using the following dosing regimen: 0, 5, 10, 20, 40, 80, 160, 160, 160 mg. Spirometry is carried out at baseline and will be repeated 60 s after each dose of mannitol. The challenge is stopped when a reduction in FEV_1_ of 15% or greater is recorded or the maximum dose has been delivered. Airway oscillometry will also be repeated post mannitol challenge.

*Skin prick testing* to common inhaled allergens is performed using the following reagents:

1. Allergopharma, Germany: histamine (positive control), saline (negative control), birch, grass mix, *Dermatophagoides pteronyssinus*.
2. Lofarma, Italy: *Aspergillus fumigatus, Alternaria alternata, Cladosporium* spp*, cat, dog*.

A weal diameter≥3 mm than a negative control is recorded as a positive test.

*Venepuncture* will be performed for full blood count (to measure eosinophil levels) and processed using Sysmex XN haematology analysers (Sysmex, UK). In addition, a serum sample will be stored at −80°C for future analysis.

*PEFv* monitoring is performed at home, morning and evening for 14 days using an e-Mini Wright Digital Flow metre (Clement Clarke International, UK).

*Asthma Control Questionnaire—5* (ACQ-5) will be administered to all participants at each visit and by telephone between CV3 and CV4.^20 21^

*Airways oscillometry* is measured in a seated position, using a nose clip, supporting the cheeks and breathing tidally for 20 s using the Tremoflo C-100 (Thorasys, Canada). A minimum of three tests are performed pre and post BDR and bronchial challenges.^22^

*MBW* is carried out using a modified Innocor LCI system (Innovision, Denmark) using the minirespiratory valve unit via an open circuit (Innocor software V.8.1).^23^ Participants breathe through a mouthpiece at a comfortable and steady rate. During the wash-in phase, a mixture of a blood soluble gas (N_2_O) and an inert insoluble gas (0.2% sulphur hexafluoride (SF_6_)) is supplied via the mouthpiece, until the concentration in their exhaled breath reaches steady state. Wash-in is achieved when the difference between inspired and expired SF_6_ is <0.2%. Participants are then switched to breathing room air and encouraged to maintain the same steady respiratory pattern (wash-out phase). During the washout, the concentration of SF_6_ in exhaled breath is recorded; a measure of 1/40 of the original concentration (0.005%) marks the end of the washout. The test is repeated three times.

*Breath sampling for VOCs* is performed using the ReCIVA breath sampler (Owlstone Medical, UK). Participants breathe tidally during the sampling period through an open mouth (6–10 min). Samples are collected onto 10 cm long steel tubes packed with adsorbent material (Tenax GR) that trap the VOCs. The tubes are then stored at 4°C until analysis (within 2 weeks).

*PExA* breath sampling is performed using PExA V.2.0 device (PExA, Sweden). The participant exhales to RV into the PExA device through the mouthpiece, then holds their breath in exhalation for 5 s before a quick deep inspiration to TLC and immediate exhalation to RV again. Particles are collected during the final exhalation. The manoeuvre is repeated until 125 ng are collected (or 30 breaths if this occurs first).^24^ In younger children, a smaller collection of 60 ng (or 15–20 breaths) are collected. Breath samples are collected onto a filter paper which is stored in a collection tube and frozen (−80°C) until later analysis.

### Outcome measures and diagnostic definitions

Outcomes from each test variable are presented in table 2.

**Table 2:** Outcome measures

	Test	Outcome measures	Established threshold for positive results
Symptoms	ACQ	ACQ-5	<0.75 good controlMinimal important difference of 0.5^20^
Tests included in NICE algorithm^5^	Spirometry	FEV_1_/FVCFEV_1_, FVC	FEV_1_/FVC<70% or below LLN
	BDR	∆ FEV_1_ or FVC following 400 µg inhaled salbutamol	≥12% (and 200 mL in adults) increase in FEV_1_ and/or FVC
	FeNO	Fractional exhaled nitric oxide levels	≥40 ppb in adults, ≥35 ppb in children
	PEFv	Daily amplitude percentage mean:((PEF_highest_–PEF_lowest_)/ PEF_mean_)x100Mean PEFv:Σ daily amplitude percentage mean/number of days	≥20% variability in Mean PEFv, provided at least 3 days data collected≥3 days with amplitude percentage mean≥20%
	BHR_mann_	Mannitol PD15	Dose of <635 mg causing 15% fall in FEV_1_
	BHR_meth_	Methacholine PD20	Dose of <0.2 mg causing 20% fall in FEV_1_
Tests of small airway function	AO	R5, R20, R5–20, X5, AX, Fres	To be established
	MBW	Lung Clearance Index, Scond, Sacin	To be established
Experimental biomarkers of small airway inflammation	PExA	Number of exhaled particles	To be established
	VOC	Mass spectrometry	To be established
Other	Skin prick tests	Number of positive tests	Weal diameter≥3 mm than negative control
	Blood test	Serum eosinophil count	>0.4×10^−9^/L

ACQ, Asthma Control Questionnaire; AO, airways oscillometry; BCT_(Mann)_, mannitol bronchial challenge test; BCT_(meth)_, methacholine bronchial challenge test; BDR, bronchodilator reversibility; FeNO, fractional exhaled nitric oxide; FEV_1_forced expiratory volume in one secondFVCforced vital capacityLLNLower Limit of NormalMBW, multiple breath washout; PEFv, peak expiratory flow variabilityPExA, particles in exhaled air; VOC, volatile organic compounds

Following completion of all study visits, the ‘expert panel’ comprising at least three physicians, including a minimum of two senior asthma physicians, meets to assign subjects a diagnosis of ‘asthma’ or ‘not asthma’. When this is not possible, participants are assigned ‘possible asthma’ or ‘insufficient evidence’.

### Asthma

The diagnosis of asthma will be determined in each case at the end of the study using a standardised format based on the following criteria:

1. Asthma definition 1: using clinical symptoms and signs consistent with asthma and all available objective evidence from conventional tests excluding novel tests. History, physical examination, ACQ, results from standard tests (spirometry, BDR, FeNO, challenges, peak expiratory flow, skin prick tests and eosinophil count results) both before and following ICS treatment are discussed, and a consensus reached. All available raw data are reviewed including flow volume loops, peak flow diaries and dose response curves.
2. Asthma definition 2: using clinical symptoms and signs alone based on information collected in a structured clerking proforma at CV1.
3. Asthma definition 3: using symptoms consistent with asthma and objective evidence of variable airflow obstruction, (determined by observation of PEF chart, spirometry pre salbutamol and post salbutamol, bronchial challenge results).

### Not asthma

Clinical symptoms of asthma are present, but there is no objective evidence from the conventional tests, either pre or post treatment with ICS.

### Possible asthma

Clinical symptoms of asthma with minimal objective evidence from the conventional tests suggestive of asthma either pre-ICS or post-ICS treatment (eg, where there might be one or more borderline test results).

### Insufficient evidence

Failure to complete sufficient visits or tests to gather sufficient objective evidence to classify participants. This group will not be used further in the analysis.

### Steroid-responsive airways disease (SRAD)

SRAD will be defined at the end of the study using a standardised format based on the following criteria:

1. SRAD definition 1: symptom responsive (≥0.5 unit improvement in ACQ-5).
2. SRAD definition 2: physiological responsive (12% (and 200 mL for adults) improvement in FEV_1_ and/or FVC, or one doubling dose improvement in provocative dose of methacholine causing 20% fall in FEV_1_ (PD_20_MCh) or mannitol causing 15% fall in FEV_1_ (PD_15_Mann).
3. SRAD definition 3: clinically responsive (clinical impression of ‘ICS responsive’ from the patient and the expert panel).

### Sample size

It is anticipated that approximately 60% of participants will fulfil the criteria of asthma^25^ and 50% will fulfil the criteria of SRAD.^26 27^ Sample size is based on the minimum of 10:1 events-to-variable ratio for logistic regression in order to avoid overfitting.^28^ With 120 (72 with and 48 without asthma) participants in the study, a multivariable logistic regression analysis, the primary analysis, can consider 5–6 variables. The coprimary outcome of SRAD (60 with and 60 without SRAD) can consider 6–7 variables. In order to account for potential dropouts (estimated 10%–20% maximum), we aim to recruit 150 symptomatic participants. Adults and children will be analysed separately, as the algorithms for diagnosis are different and the underlying pathophysiology may differ. Therefore, we aim to recruit 150 adults and 150 children.

Approximately 300 age-matched and gender-matched healthy controls will be recruited on a 1:1 ratio, to calculate reference intervals for small airways and experimental biomarkers. For non-parametric, 95% reference intervals with 90% CIs around the interval limits, a minimum of 120 participants is recommended.^29^

### Statistical analysis

This will follow a prespecified and approved statistical analysis plan. Adults (age 16+ years) and children (age 5–16 years) will be analysed separately. Where results under the age of 5 are available, these will be included in an exploratory analysis. Single variable and multivariable logistic regression analysis will be used to determine the relationship between each asthma definition and the following outcome measures: Spirometry, BDR, FeNO, PEFv, methacholine challenge, mannitol challenge, FOT, MBW, blood eosinophil, skin prick tests and specific IgE. Univariate logistic regression analysis will be used to investigate the association between each of the outcome measures and asthma. Each continuous variable will be included in its original form and dichotomised according to the predefined and rule-in test cut-points. ORs and 95% CIs will be reported. For any tests where there are repeated measures, we will use the first measurement obtained in any analysis unless otherwise stated.

Receiver operating characteristic curve analysis will be used for each continuous outcome. Cut-points for the dichotomisation of these continuous outcomes will be determined for optimising a rule-in diagnosis of asthma, by maximising sensitivity with minimum specificity of 95%. Measures of diagnostic accuracy will be reported for both the previously defined cut-points (established thresholds in table 2) and for the rule-in test cut-points.

Three approaches to creating an optimum series of investigations to predict asthma in adults and children will be used. The first approach will involve using multivariable logistic regression with model selection, for example, Least Absolute Shrinkage and Selection Operator. In the primary analysis, age will be included alongside four key variables of interest (PEF variability, BDR, FeNO and blood eosinophils). As secondary analyses, age will be included as an interaction term with the variables of interest that may have a different relationship with asthma based on age. Potential confounders such as gender, atopy, height and smoking status will be considered for inclusion in additional analyses. Further secondary analyses will consider other variables of interest, such as the bronchial challenge and small airway function data. The results of the regression analysis can be used to create a scoring system, using either continuous or categorical versions of the variables. This scoring system can then be used to define risk groups for asthma. A second approach will be to use a classification measure (such as a decision tree analysis) to determine the best way of discriminating between asthma and non-asthma participants. This will attempt to determine the best way of correctly identifying asthma using the variables of interest. The final approach will be to characterise the predictive power (area under the receiver operating characteristics (AUROC)) using bootstrapping for internal validation.

Based on the above analyses, we will consider practicalities, cost and clinical judgement to propose the optimum pragmatic diagnostic algorithm which could be tested in future studies.

A similar approach will then be used to determine the relationship between each SRAD definition and the same outcome measures.

To evaluate published diagnostic algorithms, participants will be categorised using these and then compared with our classifications from the primary outcome, asthma or SRAD. AUROC analysis will calculate the sensitivity, specificity, positive predictive value and negative predictive value of published diagnostic pathways to identify asthma. We will compare published algorithms to any new algorithms developed.

Reference intervals and repeatability coefficients will be calculated for small airways parameters, VOCs and PExA in healthy volunteers, and determine what constitutes an important change in asthma following ICS treatment. Principal component analysis and multivariable logistic regression of VOCs in exhaled breath will be used to calculate the prediction probability for asthma and SRAD.

### Ethics and dissemination

The study protocol has received a favourable opinion from the Greater Manchester East Research Ethics Committee (Ref:18/NW/0777) and granted NHS permissions by the local research office prior to commencement of recruitment. All participants or their parent/guardian are required to complete written informed consent prior to any study procedures being initiated. All children are required to complete written assent.

A manuscript with results of the primary objective will be published in a peer-reviewed journal. Further manuscripts will be written containing results of one or more of the secondary aims and objectives. Findings will also be presented at conferences. We will use Asthma and Lung UK and VOCAL (Manchester BRC PPIE group) to reach a wide audience. Data will be made available in an online data repository.

### Study status

Recruitment to the study started in mid-2019 and was significantly interrupted due to COVID-19 due to the many aerosol-generating activities in the study. Recruitment will continue until December 2027.
