# Supplementary material for: Rapid Access Diagnostics for Asthma (RADicA): protocol for a prospective cohort study to determine the optimum series of investigations to diagnose asthma using conventional and novel tests
Source: BMJ Open. 2024 Oct 26;14(10):e083908. doi: 10.1136/bmjopen-2024-083908 (PMC11529473; doi:10.1136/bmjopen-2024-083908)
Supplement: Supplementary file 1 [file bmjopen-2024-083908.reviewer_comments.pdf]

PEER REVIEW HISTORY

BMJ Open publishes all reviews undertaken for accepted manuscripts. Reviewers are asked to complete a checklist review form and are provided with free text boxes to elaborate on their assessment. These free text comments are reproduced below.

ARTICLE DETAILS

**TITLE (PROVISIONAL)**	Rapid Access Diagnostics for Asthma (RADicA): protocol for a prospective cohort study to determine the optimum series of investigations to diagnose asthma using conventional and novel tests
**AUTHORS**	Murray, Clare; Fowler, Stephen; Drake, Sarah; Wang, Ran; Durrington, Hannah J.; wardman, hannah; Healy, Laura; Bennett, Miriam; Simpson, Andrew; Barrett, Emma; Roberts, Stephen; Simpson, Angela

VERSION 1 - REVIEW

**REVIEWER NAME**	***Gilchrist, Francis***
**REVIEWER AFFILIATION**	University Hospitals of North Midlands NHS Trust, Academic Departmentof Child Health
**REVIEWER CONFLICT OF INTEREST**	Na
**DATE REVIEW RETURNED**	05-Mar-2024

**GENERAL COMMENTS**	The article by Murray et al is the protocol for the Rapid Access Diagnostics for Asthma (RADicA) trial. The protocol is well written and has sound methodology. When completed, results of this trial will fill an important void in the current available evidence regarding the diagnosis of asthma and will undoubtably be used to inform future clinical guidelines. As such, it warrants publication.<br><br>There are a few minor issues that need to be addressed:<br>1) The title is misleading as it doesn't make clear that this article is the protocol.<br>2) Information about the funding of the trial needs to be included.<br>3) Information about trial progress including planned or actual start date needs to be added.

**REVIEWER NAME**	***Vasan, Amit***
**REVIEWER AFFILIATION**	Command Hospital, Dept of Respiratory Medicine
**REVIEWER CONFLICT OF INTEREST**	Na
**DATE REVIEW RETURNED**	21-Mar-2024

**GENERAL COMMENTS**	1. Population included in >3 years and < 70 years. Children of small age group may not be able to perform forced manouvers required for spiromtery, post BDR spirometry, Mannitol and methacollin challenge test.<br>2. Oscillometry test and FENO test results will be affected when it is performed after spirometry with Post BDR testing or cha<br>3. Multiple pulmonary function tests being performed on same day

	may alter results of each other.<br>4. Acute bronchitis may mimic asthma hence duration of symptoms should be specified in inclusion criteria.<br>5. How Chronic obstructive pulmonary disease or Asthma-COPD overlap patients will be excluded from study as it can mimic asthma specially in old age group?

**REVIEWER NAME**	***Kuehni, Claudia***
**REVIEWER AFFILIATION**	Inselspital Universitatsspital Bern
**REVIEWER CONFLICT OF INTEREST**	I was an author of the ERJ practice guidelines for asthma diagnosis in schoolchildren [no other COIs indicated for reviewers].
**DATE REVIEW RETURNED**	13-Apr-2024

**GENERAL COMMENTS**	Reviewer comment<br>I have written this review together with Mari Sasaki and Beatriz Guzerro.<br><br>The authors describe an ongoing prospective cohort study, the Rapid Access Diagnostics for Asthma (RADicA) study, to determine the optimum series of clinical investigations to diagnose asthma in children and adults.<br><br>They aim to recruit patients between ≥ 3years and < 70 years (150 adults and 150 children) who are referred with suspected asthma, and conduct a series of established tests (spirometry, bronchodilator reversibility, exhaled nitric oxide, peak flow monitoring and bronchial challenge testing) and novel tests (volatile organic compounds, multiple breath washout, particles in exhaled air). The diagnostic performance of these tests using a diagnosis based on “expert panel” opinion as the reference standard, will be assessed by several statistical approaches. They also plan to compare the diagnostic performance with published algorithms, and finally also investigate the prediction performance of the novel tests. An equal number of healthy individuals, matched by age and gender to the symptomatic patients, will be recruited as controls to develop reference values of the novel tests.<br><br>The manuscript is well written and describes objectives, protocol and the analysis of the study. However, it is relatively short, and some further clarifications and updates would be most welcome to make this paper acceptable for publication. Also, it currently reads more like a protocol for an ethics application (rather technical) rather than a paper for a scientific journal.<br><br>Major comments<br>1. Overall, the text still contains some typos, which could be eradicated by careful proofreading.<br>2. Introduction: References in the introduction should be renewed. This relates particularly to diagnostic guidelines, where the European Respiratory Society clinical practice guidelines for the diagnosis of asthma in schoolchildren, developed under a UK lead, should be mentioned. Also GINA guidelines are not referenced (or I missed them).<br>3. Also references relating to the different novel tests are relatively old. It would be helpful to have at least one recent review paper for each method (FOP, VOCs, PexA etc) that summarizes the usefulness of these methods for asthma diagnosis and the justification for including them.

	4. Aims and objectives: I don't think the abbreviation SRAD has been explained before. Also the 2nd bullet point under aims is a bit technical and not totally clear (CV3)<br>5. Inclusion criteria: Please comment on the rationale of including pre-school children, below 6 years old, who are most likely not able to perform many of the lung function tests and thus, a difficult age group to diagnose as asthma. In addition, please explain if and how authors plan to specifically address this issue in the analysis stage. What are the numbers of preschool vs school-age children, and is the age-stratified sample size enough to answer the aims?<br>6. It might be important to make even clearer that the study does not include steroid naïve patients, but only asks for not having taken steroids in the 4 weeks prior to the visit.<br>7. Study setting: The sampling frame, sampling sources and sampling strategies should be explained in much more detail. I understand the study has been going on since April 2019, and recruitment is nearly complete. As test results and predictive power depend on the study population (i.e. type of setting, asthma severity, etc) a more detailed description of the recruitment will be important.<br>8. Please also explain in more detail how controls were recruited; and whether this might have introduced any bias. Do controls come from the same population as cases (except they don't have asthma)?<br>9. As most participants have already been recruited, I strongly suggest adding a table that describes relevant characteristics of those already included. As I understand BMJ open, "baseline data can be presented", but no results or conclusions should be shown in study protocol papers.<br>10. Also, information on participation rates would be welcome.<br>11. It is mentioned on P.7, that patients will be categorized as "high probability", "intermediate probability", "low probability" and "alternate diagnosis more likely" based on the initial consultation (without any objective tests). And "participants coded 'alternate diagnosis more likely' will be discussed with the supervising specialist physician and where appropriate withdrawn from the study and referred back to their GP or directly into a clinical service for further investigation". Please clarify if the patients with the final coding as "alternate diagnosis more likely" are excluded from the study. It is also unclear how the information of the probability of asthma ("high", "intermediate" and "low") is further used in the study.<br>12. Is this first clinical impression only based on history and physical examination, or also on first test results (spirometry, FeNO?).<br>13. Descriptions of the test procedures: There are many tests that are selected in both Core visit 1 and 2 in Table 1. Does this mean that these tests will be repeated, or tests that are not completed at Core visit 1 are done at 2?<br>14. Page 8: are also pre school children given dry powder inhalers, or are they directly receiving MDI and spacer?<br>15. Maybe helpful to explain the rational for planning v3 already 1 week after start of ICS – seems very short.<br>16. Could the decision process used by the expert panel be described in more detail?<br>17. P9, visit 1: is history being taken in a standardized way, i.e. using forms or questionnaires?<br>18. Concomitant medications: I had understood smokers are excluded from the study – shy should they then "withdraw from smoking prior to the visit?"<br>19. Page 10: it might be helpful to explain the reasons for the choice of allergens.<br>20. The descriptions of the tests in Table 1 are somewhat different

	to what is described in Figure 1, where only methacholine challenge is written for Core visit 2. The timing of Core visit 3 is written as "7-21 days" after Core visit 2 in Table 1 (and in the text), while Core visit 3 is described as "1-2-week response" in Figure 1.<br>21. There is also a mention of "Remote Contact" as a post-treatment visit in Figure 1, but no explanation of this in the text.<br>22. As participants are mean to perform a large number of tests, it would be helpful to indicate the time that was needed for all the measurements in each of the visits. This information should now be available as data collection is nearly finished.<br>23. Asthma definitions: The authors described the three asthma definitions that will be used on P.14. Is the only difference between Asthma definition 1 and 3 that the former uses the treatment response, but the latter does not? Please clarify the difference between these definitions and add a rationale for adopting these criteria.<br>24. Page 12: Steroid responsiveness: improvement in ACQ-5: consider regression to the mean as one explanation for improvement, given there are no controls.<br>25. Sample size: I have been taught that the sample size for prediction models relates to the number of variables that are considered to be included in the predict ion model, not the number of variables that will be contained in the final model. Consider thins and the large number of potential predictors mentioned, is I sample size large enough? Might need a short justification.<br>26. Analysis for children might need to be stratified by age, at least between preschool and school children<br>27. Statistical analysis: The authors have described PEF variability, bronchodilator reversibility, FeNO and blood eosinophils as the "four key variables" that would be in the primary analysis (P.16). Please explain how these were chosen, and why the authors do not include spirometry parameters (FEV1/FVC, FEV1, FVC) as one of the key variables, or in the further analyses.<br>28. It remains unclear how the repeated measures (taken at the different visits) will be used in the analysis.<br>29. Is there any external validation planned for the prediction models?<br>30. For the novel tests, which are not easy to perform in a clinical setting, the most relevant question might be whether they improve prediction when used in addition to the more conventional assessments. Will that be assessed, and how?<br>31. Table 2: The row heading of "AO" is missing for its outcome measures.<br>32. Abbreviations: P.6. line 56, "after early treatment (CV3)": please write the expanded form since this is the first mention of "Core visit" (or remove the abbreviation).<br>33. Please write the expanded form for "AUROC" in P.16 line 28.

VERSION 1 – AUTHOR RESPONSE

Reviewer: 1
Dr. Francis Gilchrist, University Hospitals of North Midlands NHS Trust, Keele University
Comments to the Author:
The article by Murray et al is the protocol for the Rapid Access Diagnostics for Asthma (RADicA) trial. The protocol is well written and has sound methodology. When completed, results of this trial will fill an important void in the current available evidence regarding the

diagnosis of asthma and will undoubtably be used to inform future clinical guidelines. As such, it warrants publication.

There are a few minor issues that need to be addressed:
1) The title is misleading as it doesn't make clear that this article is the protocol.

We have changed the title as suggested by the BMJ Open editor (see above) to –

Rapid Access Diagnostics for Asthma (RADicA): protocol for a prospective cohort study to determine the optimum series of investigations to diagnose asthma using conventional and novel tests

2) Information about the funding of the trial needs to be included.

Apologies this was included after the reference section. It has been moved (as per editorial suggestion) to the end of the main text.

3) Information about trial progress including planned or actual start date needs to be added.
We have added this to a section entitled "Study status" (Pg16 L501-503)

Reviewer: 2
Dr. Amit Vasan, Command Hospital
Comments to the Author:
1. Population included in >3 years and < 70 years. Children of small age group may not be able to perform forced manouvers required for spiromtery, post BDR spirometry, Mannitol and methacollin challenge test.

We agree that this will likely be the case for many small children. However, as we are also trialling a number of novel tests that include tidal breathing we have included younger children who may be able to carry out these tests, and we will then be able to compare success rates of these with the conventional tests.

2. Oscillometry test and FENO test results will be affected when it is performed after spirometry with Post BDR testing or cha

We agree with the reviewer and for this reason FeNO and oscillometry are always performed prior to spirometry and/or BDR or challenge testing.

3. Multiple pulmonary function tests being performed on same day may alter results of each other.

We agree that there is a possibility of this, and any effect is unknown. Also, it would neither be practical or reflective of clinical practice if every test were performed on a separate day. The reviewer should be assured that all tidal breathing techniques are performed prior to forced manoeuvres and reversibility is not performed on the same day as a challenge. VOC's are always collected first. The sequence of tests is the same for every individual.

4. Acute bronchitis may mimic asthma hence duration of symptoms should be specified in inclusion criteria.

All symptomatic participants have to be referred by a doctor who has a clinical suspicion that the patient has asthma. We do not specify the duration of symptoms in the inclusion criteria however we would assume that one isolated episode of acute bronchitis would not lead a clinician to believe that the patient may have asthma and such patients would not be referred to the study. We are collecting data on duration of symptoms and therefore can take this into account when analysing the data.

5. How Chronic obstructive pulmonary disease or Asthma-COPD overlap patients will be excluded from study as it can mimic asthma specially in old age group?

We can't be sure that we will exclude patients who have COPD and agree that in older patients there may be some who have Asthma-COPD overlap. However, we have attempted to exclude people with COPD, by excluding heavy smokers (>10 pack years). We plan to acknowledge that this may be a weakness of our study in the discussion of any future published papers.

Reviewer: 3
Dr. Claudia Kuehni, Inselspital Universitatsspital Bern
Comments to the Author:
Reviewer comment
I have written this review together with Mari Sasaki and Beatriz Guzerro.

The authors describe an ongoing prospective cohort study, the Rapid Access Diagnostics for Asthma (RADicA) study, to determine the optimum series of clinical investigations to diagnose asthma in children and adults.

They aim to recruit patients between ≥ 3years and < 70 years (150 adults and 150 children) who are referred with suspected asthma, and conduct a series of established tests (spirometry, bronchodilator reversibility, exhaled nitric oxide, peak flow monitoring and bronchial challenge testing) and novel tests (volatile organic compounds, multiple breath washout, particles in exhaled air). The diagnostic performance of these tests using a diagnosis based on "expert panel" opinion as the reference standard, will be assessed by several statistical approaches. They also plan to compare the diagnostic performance with published algorithms, and finally also investigate the prediction performance of the novel tests. An equal number of healthy individuals, matched by age and gender to the symptomatic patients, will be recruited as controls to develop reference values of the novel tests.

The manuscript is well written and describes objectives, protocol and the analysis of the study. However, it is relatively short, and some further clarifications and updates would be most welcome to make this paper acceptable for publication. Also, it currently reads more like a protocol for an ethics application (rather technical) rather than a paper for a scientific journal.

Major comments
1. Oveall, the text still contains some typos, which could be eradicated by careful proofreading.

We have carefully proofread the article and have corrected any typos found.

2. Introduction: References in the introduction should be renewed. This relates particularly to diagnostic guidelines, where the European Respiratory Society clinical practice guidelines for the diagnosis of asthma in schoolchildren, developed under a UK lead, should be mentioned. Also GINA guidelines are not referenced (or I missed them).

We have now referenced the ERS adult and paediatric diagnostic guidelines, along with the GINA guidelines (Refs 6,7 and 8).

3. Also references relating to the different novel tests are relatively old. It would be helpful to have at least one recent review paper for each method (FOP, VOCs, PexA etc) that summarizes the usefulness of these methods for asthma diagnosis and the justification for including them.

We agree that some of these references are quite old and have updated the article with newer reviews on the subject – References 10-17

4. Aims and objectives: I don't think the abbreviation SRAD has been explained before. Also the 2nd bullet point under aims is a bit technical and not totally clear (CV3)

SRAD is defined in the introduction Pg4, Line 105.

We agree and have changed the second bullet point in the Aims to read - Identify the best predictor(s) of response to ICS from measurements taken at baseline or after early treatment with ICS (after 1-3 weeks of treatment).

5. Inclusion criteria: Please comment on the rationale of including pre-school children, below 6 years old, who are most likely not able to perform many of the lung function tests and thus, a difficult age group to diagnose as asthma. In addition, please explain if and how authors plan to specifically address this issue in the analysis stage. What are the numbers of preschool vs school-age children, and is the age-stratified sample size enough to answer the aims?

We agree that spirometry (including BDR and challenges) is usually not performed to an adequate standard or not at all in children under 6 years. However, the children under the age of 6 are usually able to perform the oscillometry, VOC collection and multiple breath washout tests and hence the reason for inclusion. We have not prespecified the numbers of pre-school vs school age children as our sample is really a "convenience" sample; the age of the participants we recruit depends on the age of the patients we are referred. It is likely for the standard tests we will have missing data for the pre-school age group. If we recruit a disproportionally high number of 3–5-year-olds we will apply to ethics to increase our recruitment rate of older children.

6. It might be important to make even clearer that the study does not include steroid naïve patients, but only asks for not having taken steroids in the 4 weeks prior to the visit.

We agree. We have removed treatment naïve from the 'Strengths and limitations' section and added "not using ICS". In practice the majority are treatment naïve, but we did not wish to exclude those who had previously used ICS and were no longer doing so. For example, we have a number of adults in the study who had ICS prescriptions as a child but had not used them for a long time.

7. Study setting: The sampling frame, sampling sources and sampling strategies should be explained in much more detail. I understand the study has been going on since April 2019, and recruitment is nearly complete. As test results and predictive power depend on the study population (i.e. type of setting, asthma severity, etc) a more detailed description of the recruitment will be important.

We have added some additional detail to the study setting section page 6, L 164-167.

8. Please also explain in more detail how controls were recruited; and whether this might have introduced any bias. Do controls come from the same population as cases (except they don't have asthma)?

Healthy volunteers are recruited from the same local geographical area. Symptomatic participants are given flyers to pass onto healthy friends/relatives/siblings etc. We advertise with posters in the primary care practices and to staff in the local hospital who refer patients in.

We collect postcode data along with other demographics (age, gender, ethnicity) so we will be able to compare the groups.

9. As most participants have already been recruited, I strongly suggest adding a table that describes relevant characteristics of those already included. As I understand BMJ open, "baseline data can be presented", but no results or conclusions should be shown in study protocol papers.

If the editor requires this, we would be happy to include a table of the characteristics of the first 50 participants recruited. Naturally participants all meet the inclusion/exclusion criteria specified. Please specify which characteristics would be required as we would not wish to include lung function data here as these will be included in future publications.

10. Also, information on participation rates would be welcome.

Following referral of symptomatic subjects approximately 15% are screen fails and about 12 % decline to take part. However, approximately a further 15% of suitable participants then don't attend the first scheduled visit so we assume they have changed their mind.

11. It is mentioned on P.7, that patients will be categorized as "high probability", "intermediate probability", "low probability" and "alternate diagnosis more likely" based on the initial consultation (without any objective tests). And "participants coded 'alternate diagnosis more likely' will be discussed with the supervising specialist physician and where appropriate withdrawn from the study and referred back to their GP or directly into a clinical service for further investigation". Please clarify if the patients with the final coding as "alternate diagnosis more likely" are excluded from the study. It is also unclear how the information of the probability of asthma ("high", "intermediate" and "low") is further used in the study.

We confirm that participants coded 'alternate diagnosis more likely' are then withdrawn from the study and referred on to the appropriate clinical service. We have clarified this in the paper (Pg 7, L215).

The categorisation of probability of asthma is derived purely from the history and examination alone and is made by the study physician at the time of the visit. As this is often the basis of treatment decisions, when testing is not available, it will be interesting to see how well this correlates with the test results. It will be utilised in Asthma definition 2.

12. Is this first clinical impression only based on history and physical examination, or also on first test results (spirometry, FeNO?).

This is based purely on clinical history and examination.

13. Descriptions of the test procedures: There are many tests that are selected in both Core visit 1 and 2 in Table 1. Does this mean that these tests will be repeated, or tests that are not completed at Core visit 1 are done at 2?

Tests are repeated as per Table one, unless marked with* in which case may be carried out at Core Visit 1 or 2, as per Table 1 legend. We have added to the legend to try and make this clearer.

14. Page 8: are also pre school children given dry powder inhalers, or are they directly receiving MDI and spacer?

All young children are automatically given a Volumatic and Evohaler. Others have their inhaler technique assessed and will be given a Volumatic and Evohaler if the technique is not satisfactory with the dry powder device. We have clarified this (pg 8, L235)

15. Maybe helpful to explain the rational for planning v3 already 1 week after start of ICS – seems very short.

This is an exploratory part of the study; we are looking for biomarkers of early response to ICS. This is one of the secondary outcomes (pg 5 L124)

16. Could the decision process used by the expert panel be described in more detail?

Apologies. We have added more detail on this on page 12 L 364-380

17. P9, visit 1: is history being taken in a standardized way, i.e. using forms or questionnaires?

The doctor completing the history does this in a standard routine way that would be similar to any secondary care clinical practice. The patient also answers some specific symptom questionnaires so that we have the same core information on everyone. We have added this detail to pg 7 L 212.

18. Concomitant medications: I had understood smokers are excluded from the study – shy should they then "withdraw from smoking prior to the visit?"

Current smokers are not excluded. Apologies there has been an extra bullet point appeared in the exclusion criteria section. This section should read:

- **Current smokers/Former smokers with more than 10 pack-year smoking history**

We only exclude heavy current or former smokers. Smokers are asked not to smoke for one hour prior to the visit.

19. Page 10: it might be helpful to explain the reasons for the choice of allergens.

Allergens chosen are those which are the most common inhalant allergens for the UK population to be sensitised to. We have added this to pg 10, L315.

20. The descriptions of the tests in Table 1 are somewhat different to what is described in Figure 1, where only methacholine challenge is written for Core visit 2. The timing of Core visit 3 is written as "7-21 days" after Core visit 2 in Table 1 (and in the text), while Core visit 3 is described as "1-2-week response" in Figure 1.

Apologies for the error, we have redrawn figure 1 so it is accurate and matches the text and table.

21. There is also a mention of "Remote Contact" as a post-treatment visit in Figure 1, but no explanation of this in the text.

Apologies for the omission, text has been added to explain the remote contact on Page 8, L242-244

22. As participants are mean to perform a large number of tests, it would be helpful to indicate the time that was needed for all the measurements in each of the visits. This information should now be available as data collection is nearly finished.

I am uncertain as to whether the reviewer means the duration of each visit or each individual test. Most of the tests that are performed are standard physiological tests the duration of which is well known. The VOC collection takes 10-15 minutes and the PEXA collection about 10 minutes but can be quite variable from one individual to another, as we set out to collect a minimum weight of particles. The first visit is quite lengthy taking approximately 2.5-3 hours, the second visit 1.5-2hours, core visit 3 about 1 hour and core visit 4 about 2 hours. Could the reviewer confirm whether and exactly what they would like us to add to the paper and could also suggest where it is added please?

23. Asthma definitions: The authors described the three asthma definitions that will be used on P.14. Is the only difference between Asthma definition 1 and 3 that the former uses the treatment response, but the latter does not? Please clarify the difference between these definitions and add a rationale for adopting these criteria.

The key differences between Definition 1 and definition 3 (page 12) is that in Definition 3 participants must demonstrate objective evidence of variable airflow obstruction. We do have some participants in whom we label as asthma who maybe do not meet the conventional criteria for objective evidence of variable airflow obstruction (e.g. FEV1/FVC < LLN, BDR >12%, variability of >12% in FEV1 between visits). For example, Definition 1 allows a participant to be labelled as asthma with a convincing history, high FeNO, 9% BDR, PD20 0.5mg, etc. i.e. a number of borderline positive tests.

We have added to the section on outcome measures and definitions for clarity.

24. Page 12: Steroid responsiveness: improvement in ACQ-5: consider regression to the mean as one explanation for improvement, given there are no controls.

We agree with the reviewer and will consider this in any analysis.

25. Sample size: I have been taught that the sample size for prediction models relates to the number of variables that are considered to be included in the predict ion model, not the number of variables that will be contained in the final model. Consider thins and the large number of potential predictors mentioned, is I sample size large enough? Might need a short justification.

Thank you for pointing out the wording in our sample size section, we have reviewed this with our statisticians, and we agree that the wording is imprecise, and we have corrected this to "consider".

26. Analysis for children might need to be stratified by age, at least between preschool and school children

We agree that preschool children will be handled separately, their inclusion is exploratory for the novel tests we are including. We will analyse children from age 5-16 for all the conventional tests as this is the age range that most asthma diagnostic guidelines use. We have added this to page 14 L426-428 for clarity.

27. Statistical analysis: The authors have described PEF variability, bronchodilator reversibility, FeNO and blood eosinophils as the "four key variables" that would be in the primary analysis (P.16). Please explain how these were chosen, and why the authors do not include spirometry parameters (FEV1/FVC, FEV1, FVC) as one of the key variables, or in the further analyses.

We were trying to avoid parameters which were very strongly correlated with each other. For example, FEV1/FVC and bronchodilator reversibility are very strongly correlated and therefore we would include just one of these in any model for stability. We do acknowledge that we could select FEV1/FVC ratio rather than bronchodilator reversibility in future analyses.

28. It remains unclear how the repeated measures (taken at the different visits) will be used in the analysis.

We assume that the reviewer is referring to the tests which are repeated at core visit one and 2. The purpose is to repeat the novel tests so we can look at variability of these over time (PexA, VOCs, MBW, AO). Spirometry is repeated as it is required as part of the bronchial challenge test. In any analysis we will use the test the first time it is completed. We do not repeat SPT or eosinophil count. We have clarified this in the paper (Pg14, L 435).

29. Is there any external validation planned for the prediction models?

Yes, we do plan to validate any findings in a future cohort but will require further funding for this.

30. For the novel tests, which are not easy to perform in a clinical setting, the most relevant question might be whether they improve prediction when used in addition to the more conventional assessments. Will that be assessed, and how?

Yes, we agree that these tests will need to be examined not only independently but along with other tests. As asthma is a heterogeneous condition different diagnostic tests will be useful in different circumstances.

The novel tests are at different stages of development. Some need validation of the data preparation pipeline for example. Some require testing of reproducibility over time. We would calculate sensitivity and specificity for each test and will also look at correlations between novel tests and standard tests. If they appear robust, they may be included in exploratory analysis of models in future. Any finding would require further investigation and validation.

31. Table 2: The row heading of "AO" is missing for its outcome measures.

Thank you – we have added the missing heading.

32. Abbreviations: P.6. line 56, "after early treatment (CV3)": please write the expanded form since this is the first mention of "Core visit" (or remove the abbreviation).

Done.

33. Please write the expanded form for "AUROC" in P.16 line 28.

Done

VERSION 2 – REVIEW

**REVIEWER NAME**	***Vasan, Amit***
**REVIEWER AFFILIATION**	Command Hospital, Dept of Respiratory Medicine
**REVIEWER CONFLICT OF INTEREST**	Na
**DATE REVIEW RETURNED**	11-Aug-2024

**GENERAL COMMENTS**	The study protocol has been written well after including all concerns raised by reviewers. It will be a useful study to direct further research or in establishing an optimum algorithm for diagnosing Asthma.

**REVIEWER NAME**	***Kuehni, Claudia***
**REVIEWER AFFILIATION**	Inselspital Universitatsspital Bern
**REVIEWER CONFLICT OF INTEREST**	We are also conducting a study on asthma diagnosis in clinical settings.
**DATE REVIEW RETURNED**	18-Aug-2024

GENERAL COMMENTS	We think that the revised manuscript has mostly addressed the earlier concerns. There are a few minor issues that still need to be addressed.<br><br>1. Comment Nr 9 of reviewer no 3: As the authors also write that this study adopts “convenience” sampling. We thus still believe that it would be informative for the readers to understand more details of the patients who have been included up to date. This could be done by adding a table describing baseline characteristics of participants recruited up to now, by age (in groups), sex, way of recruitment, socio-economic data such as education, income, profession, area-based SES scores or whatever is available. Perhaps also duration of the disease. This can be presented together with the participation rate and perhaps a population tree in the “study status” section.<br><br>It should also be mentioned in the manuscript that the authors are planning to adjust the recruitment if there is disproportionally high number of participants in a certain age group.<br><br>2. Comment 22 of reviewer 3: The total duration of a visit for a patient should be added in study procedures. (i.e. 2.5-3 hrs for the first visit, 1.5-2 hrs for the 2nd, etc)<br><br>I did this review together with Dr. Mari Sasaki.

VERSION 2 – AUTHOR RESPONSE

Reviewer: 2
Dr. Amit Vasan, Command Hospital, Command Hospital Lucknow
Comments to the Author:
The study protocol has been written well after including all concerns raised by reviewers. It will be a useful study to direct further research or in establishing an optimum algorithm for diagnosing Asthma.

Response: No response required; thank you for the comment.

Reviewer: 3
Dr. Claudia Kuehni, Inselspital Universitatsspital Bern
Comments to the Author:

We think that the revised manuscript has mostly addressed the earlier concerns. There are a few minor issues that still need to be addressed.

1. Comment Nr 9 of reviewer no 3: As the authors also write that this study adopts “convenience” sampling. We thus still believe that it would be informative for the readers to understand more details of the patients who have been included up to date. This could be done by adding a table describing baseline characteristics of participants recruited up to now, by age (in groups), sex, way of recruitment, socio-economic data such as education, income, profession, area-based SES scores or whatever is available. Perhaps also duration of the disease. This can be presented together with the participation rate and perhaps a population tree in the “study status” section.

It should also be mentioned in the manuscript that the authors are planning to adjust the recruitment if there is disproportionally high number of participants in a certain age group.

Response 1: The editor has agreed that the addition of baseline data for participants recruited so far is beyond what would normally be expected in a study protocol paper and therefore we have not provided this. With regard to the question about changing the manuscript to state that we would adjust the recruitment if there were a disproportionally high number of participants in a certain age group, however we do not believe this to be necessary. We had responded to an earlier comment that "if we recruit a disproportionally high number of 3–5-year-olds we will apply to ethics to increase our recruitment rate of older children" and we assume that this is what the reviewer is referring to. This is a hypothetical situation, we have no reason to suspect that this would be the case and thus do not feel that it is necessary to alter the manuscript accordingly.

2. Comment 22 of reviewer 3: The total duration of a visit for a patient should be added in study procedures. (i.e. 2.5-3 hrs for the first visit, 1.5-2 hrs for the 2nd, etc)

Response 2: We have added the approximate visit duration times to Table 1 – the study schedule, as this seemed the most efficient.

I did this review together with Dr. Mari Sasaki
